# Study of the Efficiency of Fog Computing in an Optimized LoRaWAN Cloud Architecture

**DOI:** 10.3390/s21093159

**Published:** 2021-05-02

**Authors:** Jakub Jalowiczor, Jan Rozhon, Miroslav Voznak

**Affiliations:** Faculty of Electrical Engineering and Computer Science, VSB-Technical University of Ostrava, 17. listopadu 2172/15, 708 00 Ostrava, Czech Republic; jan.rozhon@vsb.cz (J.R.); miroslav.voznak@vsb.cz (M.V.)

**Keywords:** fog computing, cloud computing, internet of things, simulation, network architecture, LoRaWAN

## Abstract

The technologies of the Internet of Things (IoT) have an increasing influence on our daily lives. The expansion of the IoT is associated with the growing number of IoT devices that are connected to the Internet. As the number of connected devices grows, the demand for speed and data volume is also greater. While most IoT network technologies use cloud computing, this solution becomes inefficient for some use-cases. For example, suppose that a company that uses an IoT network with several sensors to collect data within a production hall. The company may require sharing only selected data to the public cloud and responding faster to specific events. In the case of a large amount of data, the off-loading techniques can be utilized to reach higher efficiency. Meeting these requirements is difficult or impossible for solutions adopting cloud computing. The fog computing paradigm addresses these cases by providing data processing closer to end devices. This paper proposes three possible network architectures that adopt fog computing for LoRaWAN because LoRaWAN is already deployed in many locations and offers long-distance communication with low-power consumption. The architecture proposals are further compared in simulations to select the optimal form in terms of total service time. The resulting optimal communication architecture could be deployed to the existing LoRaWAN with minimal cost and effort of the network operator.

## 1. Introduction

The vast popularity of the Internet of Things (IoT) has caused a rapid increase in the number of connected devices through new technologies and market trends. These new technologies are applied in many different fields, including e-health [1], surveillance [2], security [3], and many others. As the number of connected IoT devices grows, it is essential to develop specific technologies to provide wireless IoT connectivity. Low-Power Wide-Area Network (LPWAN) technologies are sufficient solutions for IoT applications that require urban-wide coverage. These technologies allow for long-range transmission with low power consumption and include technologies, such as LoRa [4]. LPWAN technologies most usually adopt the cloud computing paradigm. The cloud computing paradigm provides resources (computing, storage, services, applications) through the Internet. Its advantages combine reduced management, easy resource allocation based on usage, and the pay-as-you-go cost model [5].

Although the exact predictions for future expansion of connected IoT devices vary considerably, the increasing trend is clear. As the number of IoT devices increases, the demands for speed and data volume are higher. This situation can lead to a certain threshold when cloud computing is not an effective solution nor sufficient for specific IoT use cases. The fundamental limitation of the cloud computing paradigm is a significant distance between the cloud and end-nodes that negatively affects latency, jitter, and location-awareness. The recent state-of-the-art in IoT network architectures shows that the current trend is a shift from the cloud computing paradigm to fog computing. While cloud computing, in some situations, cannot keep pace with the growing volume and speed of transmitted and processed data, the fog computing paradigm can increase efficiency, as well as achieve low latency and location-awareness [6]. Fog computing combines cloud computing with edge computing [7] and offers the benefits of both. While the majority of processing is done by edge devices that additionally operate as data filters, a cloud is presented to perform less urgent or more complex computing. From that perspective, the fog computing paradigm can be seen as an extension of the cloud computing paradigm in applications and domains that the cloud paradigm does not support. Fog computing can better adapt to the needs of end-nodes because of location-awareness.

The primary motivation to study and propose the solution based on LoRa and LoRaWAN is the possibility of long-range communication [8], low energy consumption [9], and many existing deployments of the LoRaWAN network in a large number of locations. A network operator only updates individual components of an existing network to support fog computing because LoRaWAN technology is widely deployed. The end-devices and the hardware can remain unchanged. That makes fog computing features available at a low cost. An example can be the campus of the VSB-Technical University of Ostrava, where LoRaWAN is already deployed, and we want to add fog computing optimizations for further research.

Imagine a company that already uses LoRaWAN to collect specific information regarding their business within a production hall. The company has a web application that is available to observe individual information or control activities based on the obtained data. The solution proposed in this study could extend available applications with many valuable features, which can be addressed by providing local computing and storage resources. The company can define which data are private, and this information is subsequently transferred to the corresponding fog node. Once the fog node has this information available, private data can be processed and stored locally, while the other data can be transferred to the cloud. Fog nodes can implement a connection failure detection method. When a connection failure is detected, all of the resources are computed and stored in the fog layer until the connection is re-established. In this manner, data received during the connection failure are not lost. Lower latency of fog computing as compared to cloud computing plays a key role in applications that require a faster response. These are just a few examples of relevant use-cases of the fog computing paradigm, but there are many more.

In the present study, we propose three different network architectures that are based on LoRaWAN and the fog computing paradigm. However, the question arises as to which of the proposed architectures is optimal. This can be determined by comparing the proposals according to several characteristics. The paper aims to compare the proposed architectures from the perspective of service time. Therefore, we will compare the proposed architectures in simulations that are based on queuing theory.

The paper is structured, as follows. The Section 1 introduces the research area of this work. The existing studies are summarized in the Section 2. The next section discusses general fog computing architecture, together with expected features. The Section 4 provides a brief insight into the standard LoRaWAN architecture components. The Section 5 describes the proposed optimized fog computing architectures. The Section 6 describes simulation models. The following section summarizes and discusses the results. The final section is devoted to a summary of conclusions.

## 2. State-of-the-Art

This section summarizes related works and reports the current state of fog computing applications in IoT and LoRaWAN.

ARM, Cisco, Dell, Intel, Microsoft, and Princeton University funded OpenFog Consortium in 2015 to define an open fog computing architecture. According to the definition that is presented in the document OpenFog Reference Architecture [10], fog computing is an extension of the regular cloud-based computing paradigm, where all the advantages of the cloud should be preserved. An overview of fog computing can be found in [11,12,13]. As mentioned in [14], time-sensitive processing can be handled at the edge of a network, while other traffic can be processed in a cloud. A variety of research opportunities in the field of fog computing are in [15]. In [16,17], the authors analyzed and described the security and privacy issues of fog computing in IoT.

Most of the authors in their studies deal mainly with the implementation of efficient IoT network infrastructures in terms of reduced energy consumption or the adjustment of radio parameters, such as in [18,19,20]. Significantly fewer authors deal with a change of communication between individual components of network architecture, acceleration of data exchange, or transfer of resources, although there is a lot of room for improvement.

Gia et al., in [21], introduced system and gateway architecture, followed by the experimental results of a fog computing case study in healthcare IoT for ECG feature extraction. The performance simulation of fog computing architecture intended for smart cities was presented in [22]. The proposed architecture reduces latency and improves energy provisioning. Gupta et al. in [23] proposed iFogSim software simulation of IoT and fog environments to evaluate resource management policies that consider their effect on latency, energy consumption, and other properties. In [24], the authors used iFogSim to evaluate the proposed QoE-aware application placement policy in fog computing environments.

A comparative study of LPWAN technologies in [25] showed that, while Sigfox and LoRa technologies have better assumptions for applications requiring longer battery lifetime, higher capacity, and lower cost, NB-IoT offers advantages in the case of latency and QoS. However, LoRa end-devices can operate in class C (continuous) mode to handle bi-directional communication with lower latency, but with higher energy consumption. The authors in [26] described the design and experimental deployment of LoRaWAN infrastructure and the deployment of fog computing nodes at the smart campus at the University of A Coruña. However, the paper primarily focuses on the planning of signal radiation and coverage. In [27], the authors introduced LoRaWAN architecture with autonomous base stations and provided an example of its application in areas with limited internet access. The authors proposed master and slave base station architecture, in which the master base station acts as a central point for many slave base stations and, in this manner, the internet connection for slave base stations is not required. The authors in [28] developed a message replication mechanism for LoRaWAN to improve emergency communication reliability. That is done by the intentional redundancy of messages, because one physical end-device uses different spreading factors to send identical traffic. The presented mechanism reduces the loss of emergency messages and the average transmission time. A smart IoT irrigation system based on LoRaWAN technology that adopts the fog computing paradigm can be seen in [29]. The designed network architecture contains three layers. Sensors in the IoT node layer communicate with LoRaWAN gateways presented in the fog computing layer. The fog computing layer includes a central LoRaWAN server shared between gateways to provide fog computing services. The remote service layer is able to store collected data or exchange it with external services. The results of this study can be used as guidelines to design a smart irrigation system.

## 3. General Fog Computing Architecture

**Figure 1:**
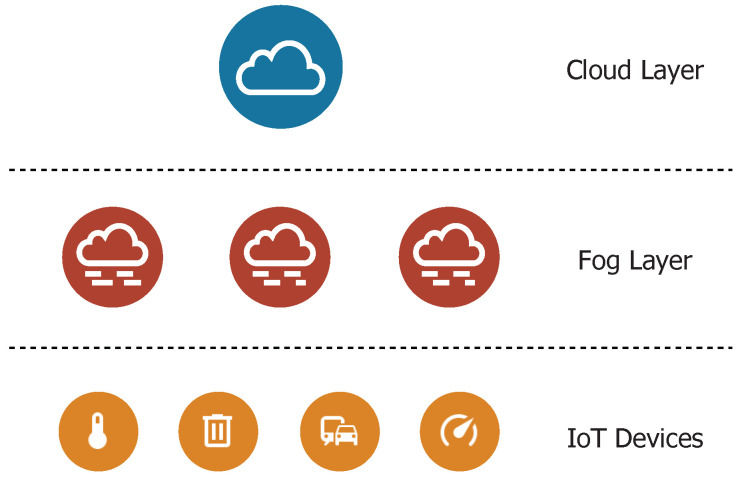
Three-layer fog computing architecture model.

Because the proposed architecture adopts fog computing, it contains three layers. Figure 1 presents the three-layer architecture model. Individual IoT end-nodes are located in the bottom layer. The fog layer is presented in the middle of the model and it includes fog nodes that are intended for partly autonomous decision-making. The fog nodes operation is mainly affected by the received traffic and collaboration with the cloud layer. The cloud layer is the upper layer in the model and includes a cloud server [8].

The following properties must be preserved to adopt the fog computing paradigm:

- Location-awareness—because of the closer distance to end-nodes, the fog layer can adapt its features according to diverse end-nodes requirements.
- Efficiency—results from the collaboration between the fog and the cloud layers. If needed, data processing and storage can be moved from the cloud to the fog layer and inversely. This change can happen in many circumstances, for example, during unavailable resources.
- Lower latency—can be reached through computing at the fog layer. The lower latency adds support for applications that require a faster response, for example, alarm or e-health systems.

The properties that are mentioned above are the main reasons for the engagement of fog computing in the IoT, and their preservation is essential.

## 4. Standard LoRaWAN Architecture

The openness of LoRaWAN protocol allows deployment and management of privately owned LoRaWAN networks, where the owner can modify individual components of the network architecture. The following list summarizes standard LoRaWAN network elements [4].

- **End-devices**—are sensors or actuators capable of wireless communication with gateways using LoRa RF modulation. The end-devices are mostly battery-powered.
- **Gateway**—is presented between a LoRaWAN network and an IP network such as Ethernet or Wi-Fi, and it performs bridge functionality. The gateway serves as an input to a LoRaWAN network for end-devices, transforms RF packets into IP packets, and forwards them to the associated network server.
- **Network server**—is a central point of the network, which performs network management. Among other features, the network server performs the filtration of duplicate or unnecessary packets and handles adaptive data rate schemes. It routes packets from end-devices to the associated application server.
- **Application server**—handles all of the application layer payloads. The application server resolves required operations with received data, such as decryption, database storage, or visualization through an associated web-based user interface.

**Figure 2:**
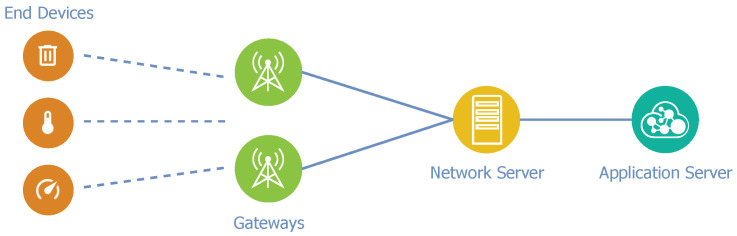
Standard LoRaWAN architecture.

In the standard LoRaWAN network architecture that is depicted in Figure 2, the gateways are the closest section of the network from the end-device perspective. Each gateway acts as a passive area of the network, because it does not perform any computational operations. The gateway receives data from end-nodes and then converts packets into a form suitable for further transportation and processing. Subsequently, converted data are routed to the associated network server. The application server performs data processing, computing, and storage. The description shows that the computing model in LoRaWAN corresponds to the cloud computing paradigm.

## 5. Proposed Network Architectures

The basic idea of the present research is as follows. If the LoRaWAN basic elements are affected by specific changes, they can be transformed into the fog computing paradigm supporting architecture. The main challenge is end-to-end encryption of the application payload, which is encrypted between the end-device and application server. Because of that, the gateway itself is not able to access decrypted data and, as a result, it cannot provide data processing and storage.

Two session keys stored in the end-devices memory:

- **Network Session Key (NwkSKey)**—the network server and the end-device use this key to evaluate and verify the message integrity code (MIC). The MIC ensures the integrity of data in data messages. It is stored in the end-device and the network server after successful activation.
- **Application Session Key (AppSKey)**—this key is designed for the decryption and encryption of the application payload. It is stored in the end-device and the application server after successful activation.

The application payload is not integrity protected and, therefore, the risk arises that the network server may change the content of a data message. However, network servers are commonly considered to be trustworthy. Both session keys are specific for each end-device. More information about the keys and decryption is presented, for example, in [30].

The following is a description of the three different network architecture proposals. We subsequently compare these proposals to determine which is optimal in terms of the queuing theory.

### 5.1. Architecture A

The proposed architecture A is based on the idea that all standard LoRaWAN network elements can be integrated into a single unit, called fog gateway. In this manner, each gateway, in addition to receiving, converting, and relaying packets, performs the functions of the private network and application servers. This adjustment could lead to a faster response to current conditions according to actual data.

**Figure 3:**
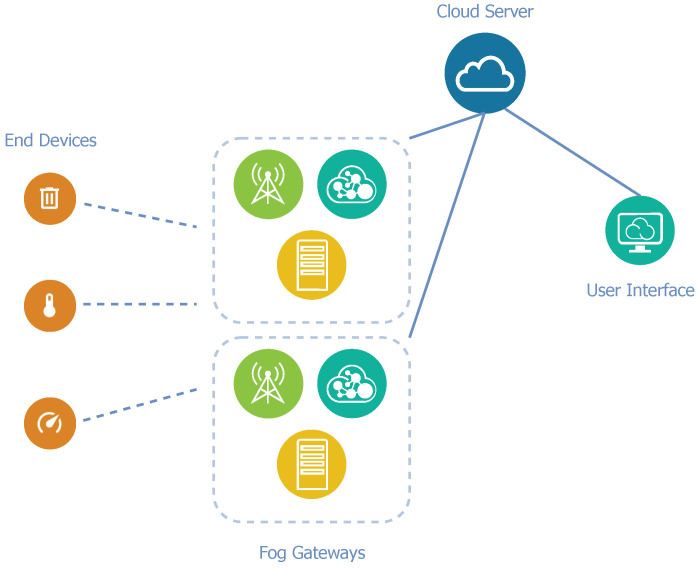
Diagram of network architecture A.

However, fog processing is not always necessary, and sometimes it is not even possible. Especially when a great amount of data is being transferred, a disproportionate load could arise when considering the limited computing resources of fog gateways. To solve the problem, a remote application cloud server is presented in this architecture as an addition to fog gateways. For the entire LoRaWAN network, only one cloud server is available for all of the fog gateways. This cloud server provides a web-based graphical interface and APIs to manage fog gateways, devices, and applications. We can see a diagram of architecture A in Figure 3.

### 5.2. Architecture B

Architecture B applies a slightly different view on optimization. Unlike architecture A, the proposed architecture B does not require the integration of the network and application servers into each fog gateway, but they are only integrated into one gateway in the network. The remainder of the gateways in the network have identical functions as gateways in the standard LoRaWAN network, and they can only receive, convert, and relay packets. For the correct operation of such a proposed architecture, it is necessary to involve a certain communication model. The master–slave model comes into play because a single complex node controls multiple nodes with limited logic.

Master–slave is a communication model in which a single node (master) controls multiple slave nodes and mediates the communication between them. These master and slave nodes are gateways in this case. The slave gateways operate in the standard LoRaWAN gateway mode. In this mode, the slave gateway transfers each received message to the master gateway. The master gateway integrates the network and application servers to decrypt the payload and control packet relaying by the slave nodes.

**Figure 4:**
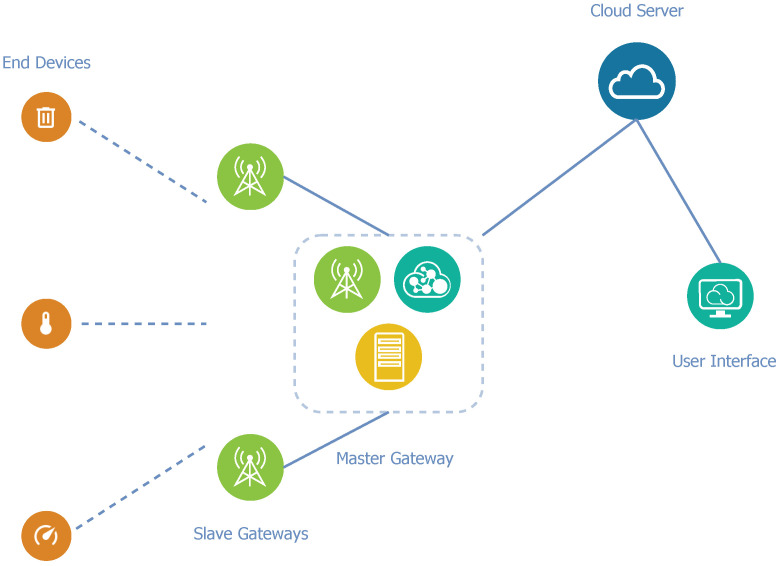
Diagram of network architecture B.

The remote cloud server is a part of the architecture due to the limited computational and storage resources of the fog gateway. The cloud server is connected to the master gateway via the Internet and it is used to store long-term data. A diagram of architecture B can be seen in Figure 4.

The elimination of the need for an Internet connection for each gateway is the main advantage of this architecture. An Internet connection is necessary while applying standard LoRaWAN architecture, because the network and application servers are situated in a cloud. Only the master gateway may be connected to the Internet in architecture B, as it communicates with the remote cloud server.

### 5.3. Architecture C

Architecture C applies an entirely different principle than previous architecture optimizations. Instead of reallocating individual elements of the standard LoRaWAN architecture, the main change here is communication between a gateway and the application server. In this manner, architecture C does not differ from the standard LoRaWAN architecture in terms of structure.

**Figure 5:**
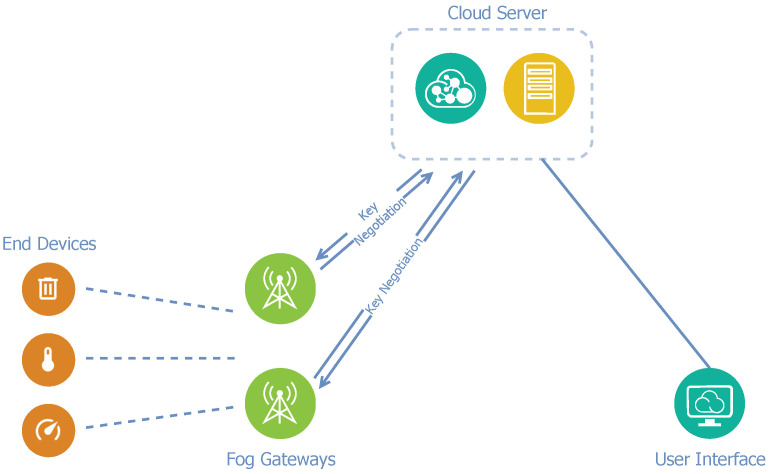
Diagram of network architecture C.

As already mentioned, because of end-to-end encryption, a LoRa gateway itself does not know the session keys and it cannot access the decrypted payload since the application server performs the decryption. The core concept of optimization is the negotiation between a specific fog gateway and the application server to obtain and, subsequently, store the necessary keys for the individual end-device in the local secured database. Thereby, a fog gateway only communicates with the application server if it does not yet have the required session keys for a specific end-device or in the case of need. Thus, interaction with the application server is minimized. A significant benefit of this architecture design is that the network operator can deploy the fog gateway to the existing LoRaWAN network without modifying the cloud layer. Figure 5 presents a diagram of architecture C.

## 6. Comparisons in Terms of Queuing Theory (Service Time)

Because an IoT network can be viewed as a particular queuing system, it is possible to use queuing theory and compare the proposed architectures in terms of service time. Individual messages from end-devices can be seen as requests to be served. The LoRa gateway, network server, and application server are queuing nodes. Assuming that the gateway can receive up to eight LoRa messages simultaneously with different SFs on different channels [31], the queuing node representing the gateway has eight servers that can serve requests concurrently. To establish the same conditions for each of the proposed architectures, we assume that all end-devices fall into class A, use the ABP activation method, and send messages without the need for acknowledgement.

Because the considered physical layer (LoRa) is the same for all architectures, the simulation is mainly focused on the processing of data by individual elements of the architecture. For correct simulation of data processing, it is necessary to model the behaviour of an individual end-device while sending messages in a certain manner. It is often the case that IoT devices, such as sensors, trackers, and other similar devices, send their data periodically in specific time intervals. Aggregated traffic from a large number of the mentioned IoT devices can be seen as a superposition of deterministic point processes. If these individual point processes are considered to be independent, and the individual devices generate their messages independently, this operation can be modelled according to the Poisson process. We can describe aggregated traffic using interarrival times, which are the intervals between end-device messages from a LoRa gateway perspective. Because of the presence of this periodicity during message transmission by particular end-devices, an error element is involved in the Poisson approximation. The authors of publication [32] assumed that, in the case of lower loads with an average use of 0.55 in a comparison of nD/D/1 and M/D/1 systems, the difference between them was negligible. However, messages and their payloads have varying sizes if we consider the real environment of an IoT network. Thus, service time is not deterministic, but variable, and it corresponds to an exponential distribution. Therefore, the individual queuing systems in the present study are modelled according to the M/M/n model, where *n* is selected according to the specific simulated part of the network.

The problem at this point is that generally valid values for the required service times do not exist, but they can be obtained through experimental measurement on real hardware components. Of course, the results depend on the computational performance of these components, but, assuming identical hardware for each of the proposed architectures, the simulation results should not possess any distortions.

### 6.1. Experimental Measurement

To obtain the service times for the individual elements of each architecture, a series of experimental measurements were conducted as a part of this study. A Raspberry Pi 4 Model B with 2 GB of RAM and Raspbian OS was used as hardware for the LoRa gateway. We selected a virtual machine with 2 GB RAM and OS Debian, which offered sufficient computational power, to accomplish the function of the network and application servers. Execution time was measured from the time the message was received by the gateway because the time of message propagation in the radio environment was not essential for these experimental measurements. The results were determined at a 95% confidence level.

For the proposed architectures A and B, the gateway (packet forwarder) execution time ($T_{pktfwd}$) was measured until the message was forwarded to the network server that continued with message processing. The network server’s execution time was measured from the time that the message was received by this server until the time the message was processed and forwarded to the application server. The execution time by the application server was measured from the time that the message was received until the time the decrypted payload was added to a database. The experimental measurements were conducted for 1000 messages. We subsequently calculated the average time. Table 1 shows the results of these experimental measurements.

The proposed architecture C applies a different method, since the packet forwarder is specially modified to perform fog computing operations. In this case, the packet forwarder was designed to forward the data to a network server and additionally decode the messages and decrypt the payload. Therefore, it is necessary to add the execution times of individual fog computing functions that were performed by the gateway, i.e., message decoding ($T_{decode}$), payload decryption ($T_{decrypt}$), and subsequent storage of the payload to the database ($T_{database}$), to the experimental measurement result of the execution time by the packet forwarder.

$$\begin{array}{cc} \; & T_{fog}=T_{decode}+T_{decrypt}+T_{database}\\ \; & T_{total}=T_{pktfwd}+T_{fog} \end{array}$$

The above equations can be used to obtain the total message execution time that is necessary to perform fog computing functions related to proposed architecture C. As in the previous case, a Raspberry Pi 4 Model B was used as gateway hardware for the experimental measurements. One thousand measurements of the individually mentioned functions were gradually performed. The algorithm generated a pseudo-random string of at least 20 characters for each decoding and decryption process measurement. The decryption process has been described in detail, for example, in [30]. The storage of metadata and payload to the database was subsequently measured to obtain the final time component. Table 2 shows the detailed results of these experimental measurements.

Table 3 contains the average total times that are necessary to process messages by the fog gateway. These times were determined according to the equations mentioned above.

### 6.2. Simulation Preface

In the previous step, we obtained the required execution times for each component of the architecture, and it was then possible to proceed to the simulation itself. For the simulation, we used the graphical programming environment Simulink, which is an extension of the software tool Matlab. The Time-Based Entity Generator block generated individual service requests for each of the simulated architectures. The integration time corresponded to the Poisson distribution, where the mean, for the sake of the simulation, was set to 0.1. We selected this value to keep the packet forwarder usage low (around 20%) and, therefore, the resulting time was not affected by blocking requests in the queue. We only used the data privacy value as input in deciding whether to select between fog or cloud computing to reduce the complexity of the simulation. In the case of a private message payload, the message was processed by the fog gateway. In the case of a public message payload, the message was transferred to the public cloud for further processing. All of the simulations were performed for 10,000 messages.

### 6.3. Architecture A Simulation

**Figure 6:**
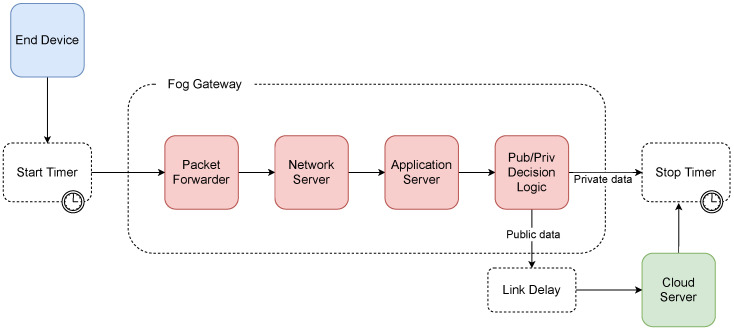
Diagram of the simulation model of architecture A.

In the simulation of proposed architecture A, the n-server block representing the packet forwarder on the gateway side was included first. Here, the average service time was set to 0.16 ms and the number of lines (*n*) to 8. Subsequently, the request was passed to the infinite server (*n* = *∞*) block, which represented a network server with an average service time of 194.62 ms, followed by a chained infinite server block, which represented an application server with an average service time of 15.16 ms. After processing by these chained queuing systems, the application server evaluated whether the data were private. If true, the current processing time was stored, and the simulation cycle ended. If it was not private, the application server forwarded the message to a remote cloud server. Thus, a delay on the line with a length of 0.5 ms was added to the total execution time. Because the fog gateway forwarded the data to the cloud server after processing by the network and application servers, the cloud server only inserted the data into the database. Thus, its average execution time was brief and set to 7 ms, according to the results of the experimental measurements. When the decrypted payload was stored in the database, the total execution time was then saved, and the simulation cycle ended. A diagram of the simulation model can be seen in Figure 6.

Because architecture B is only a variation of architecture A, in which the network and application servers were not implemented in each gateway, but only in a master gateway, it was not necessary to perform a simulation for this architecture. The resulting execution times only differed in a line delay of an expected time of 0.5 ms during transmission between the slave and master gateways, which was not needed in architecture A.

### 6.4. Architecture C Simulation

For the proposed architecture C, the n-server block representing the packet forwarder was the first block included in the simulation. The average service time of this block was set to 0.16 ms and the number of lines to 8. Subsequently, the data were evaluated as to whether they was private or public. In the case of private data, the processing request was routed to the chained infinite server blocks for decoding, decryption, and insertion into the local database. These blocks used average service times $T_{decode}$, $T_{decrypt}$, and $T_{database}$, respectively. This step ended processing of the message in the case of private data, the total execution time was subsequently saved, and the simulation cycle ended.

**Figure 7:**
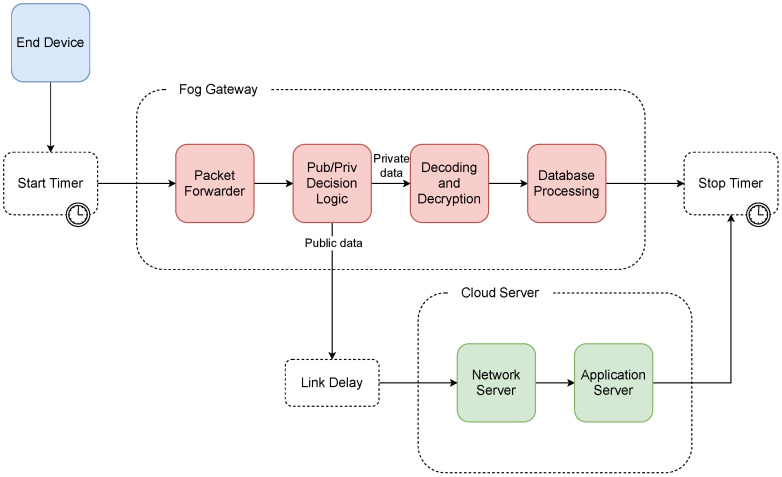
Diagram of the simulation model of architecture C.

In the case of transferring public data, the message was forwarded to a cloud in which a link delay of 0.5 ms was included. The cloud consisted of the network and application servers with average service times of 194.62 and 15.16 ms, respectively. When processing by these queuing systems was completed, the total execution time was then saved, and the simulation cycle ended. A diagram of the simulation model for proposed architecture C can be seen in Figure 7.

## 7. Results and Discussion

**Figure 8:**
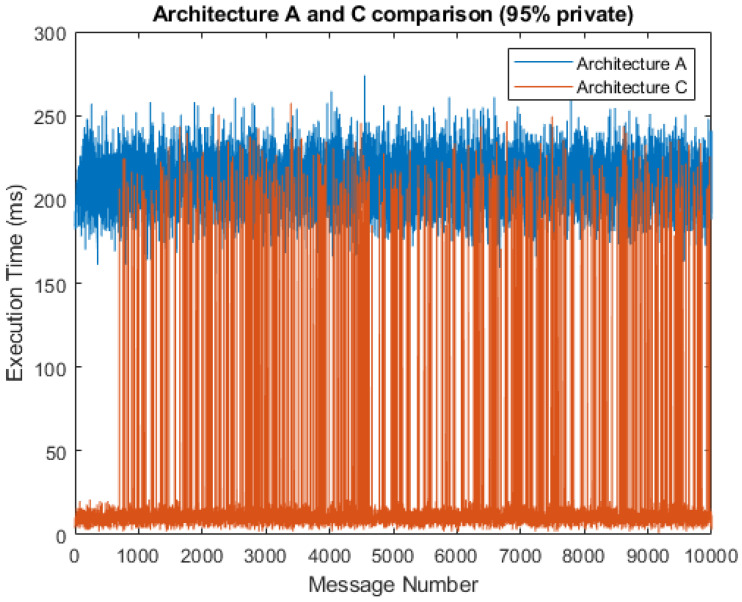
Comparison of architectures A and C (95% private messages).

Figure 8 shows a comparison of the total service times that result from the simulation with a 5% probability of public messages. The chart shows that architecture C had a much shorter total message execution time, with the average service time being 19.44 ms as compared to 209.96 ms in architecture A. This, of course, only applied when private data predominated and primary data processing was done by the fog gateway, which is the principle of fog computing and the type of solution proposed in this study. Otherwise, the execution times for architecture A and C were almost identical. We can observe significant jumps in the execution time for architecture C, illustrating the transmission of messages to a public cloud server, where messages are then further processed. If the simulation is limited to only fog gateway processing, these jumps do not occur.

**Figure 9:**
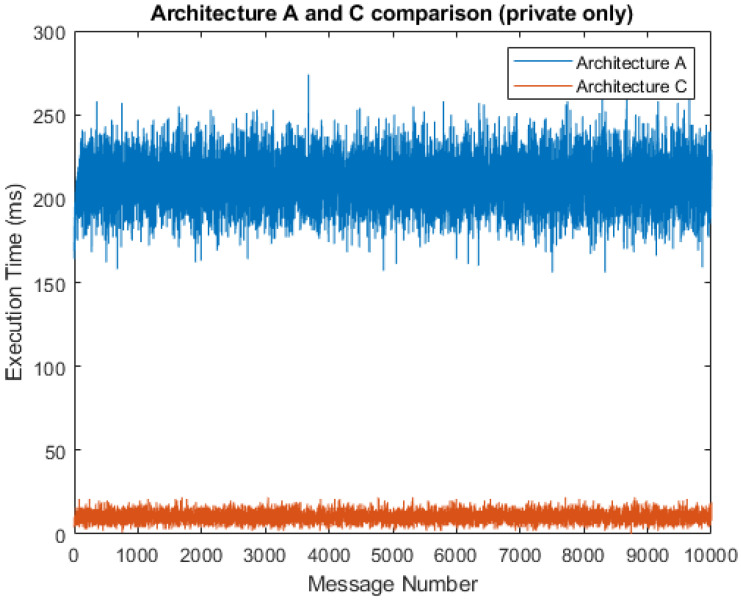
Comparison of architectures A and C (100% private messages).

A simulation without transmission to the cloud server can be seen in Figure 9. In this case, the average service time was 10.28 ms for architecture C when compared to 209.81 ms for architecture A. This is the best-case execution time (BCET), as we are discussing the shortest time for any possible combination of inputs. The chart shows that the BCET for architecture C is lower because of the absence of significant jumps, since the data do not need to be processed by the network and application servers. For architecture A, the BCET is similar to the 5% probability of public messages. The similarity is a result of the presence of the network and application servers in each fog gateway. In this manner, data processing is mainly performed by the fog gateway, and the cloud server only performs insertions into the local database. The worst-case execution time (WCET) occurred when all the data were set to public and, in this manner, the fog computing operations remained idle. In this case, the WCET of architecture C was 210.41 ms, a similar result to the WCET of architecture A with 217.78 ms.

**Figure 10:**
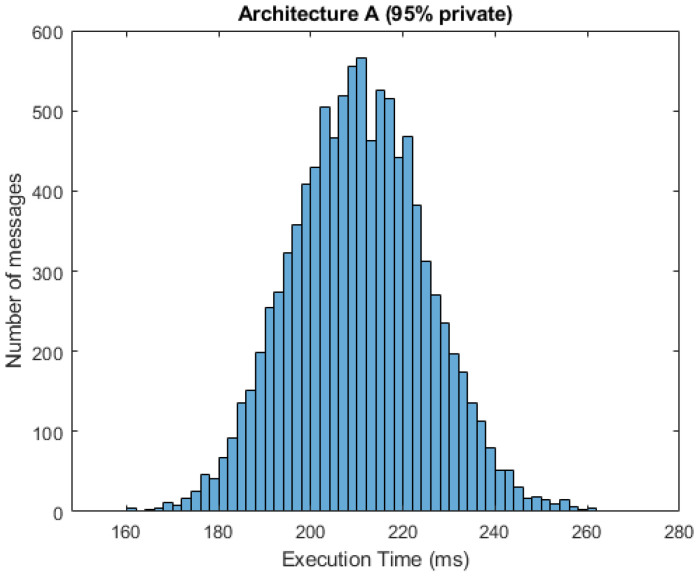
Histogram for architecture A (95% private messages).

**Figure 11:**
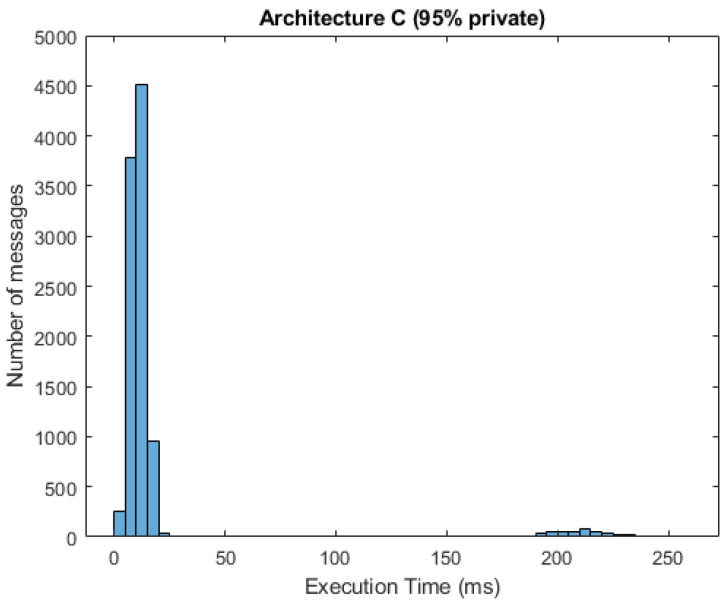
Histogram for architecture C (95% private messages).

**Figure 12:**
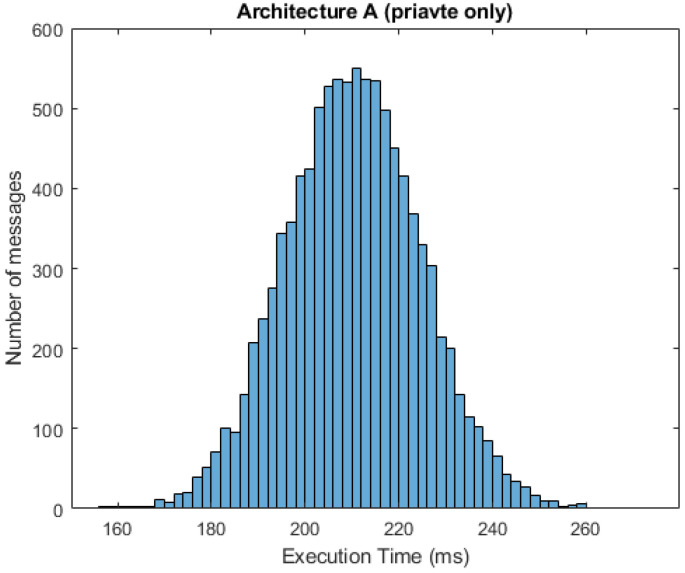
Histogram for architecture A (100% private messages).

**Figure 13:**
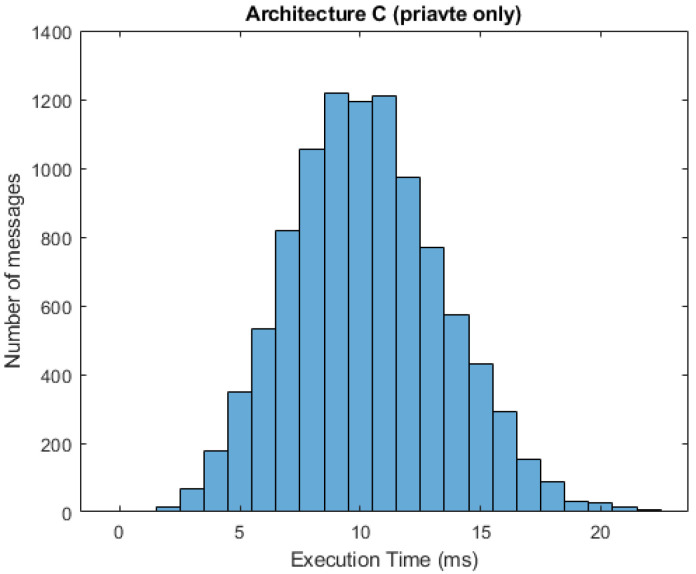
Histogram for architecture C (100% private messages).

In Figure 10 and Figure 11, we can see respective histograms for architecture A and architecture C, with a 95% probability of private messages. A comparison of these histograms shows that while the total service time values for architecture A were not scattered significantly, the histogram for architecture C was dispersed, and the variance of these values was much higher as a result of the already mentioned difference in private and public processing. In Figure 12 and Figure 13, the histograms show the simulations for architectures A and C with a 100% probability of private messages. These histograms imply that, while the spread of the histogram for architecture A was almost the same in both cases, the variance of values that presented in the histogram for architecture C with a 95% probability of private messages was much higher than the case of only private processing. The simulation results summary of the individual proposed architectures can be seen in Table 4.

We can choose a time base T corresponding to the BCET of architecture C (10 ms) to approximate the resulting service times for any hardware solution. Subsequently, we can express the resulting times as a multiplication of the time base. Table 5 shows a summary of the approximated resulting times obtained by the simulations. The constant $T_{link}$ is the link delay during transmission between the slave and master gateways. As we can see, proposed architecture C achieved the best results in all cases. However, the table also indicates that the gap between the BCET and WCET for this architecture is wide. The simple rule is that the more public data are transferred on the network, the closer the total execution time will be to the WCET.

If we consider exclusive processing and storage of messages in the fog layer (only private messages), the resulting service time varies, depending on the current workload. As mentioned earlier, this is highly affected by the specific hardware components of the fog gateway. However, for the completeness of the study, the following Table 6 contains the simulation results achieved under different workloads for 100,000 messages.

The resulting order of the architectures remained the same, as can be seen from the results. Architecture C outperformed the other architectures, even though the workload is heavy. That is caused by the omission of network and application servers in the case of fog computing in architecture C.

## 8. Conclusions

The cloud computing paradigm is not able to satisfy the requirements to maintain specific IoT applications and location awareness. In contrast, the fog computing paradigm utilizes a different strategy. Instead of focusing all of the computation and data storage into the cloud layer, it places the fog layer between the end-devices and the cloud. In the present paper, we proposed three IoT network architecture schemes adopting the fog computing paradigm into the LoRaWAN cloud architecture. To select the optimal of the three proposed, we used a software simulation tool to apply queuing theory and compare the proposed architectures in terms of service time. We observed significantly shorter total execution times for proposed architecture C from the achieved simulation results. However, this was not the case for WCET, in which the execution times produced results that were similar to the other proposed architectures.

In future research, we would like to extend the study presented in this paper and compare the individual proposed architectures in terms of functional properties. Once we have all of the results, we can select a suitable architecture proposal for implementation.

## Figures and Tables

**Table 1:** Execution times of the components.

Component	Average Execution Time (ms)
Packet forwarder (gateway)	0.16
Network Server	194.62
Application Server	15.16

**Table 2:** Descriptive statistics of the experimental measurement results (miliseconds).

Time	Mean	St.Dev.	Min.	Max.	Med.	Q1	Q3	Skew.	Kurt.
$T_{decode}$	1.95	0.90	0.83	5.08	1.65	1.54	1.91	2.33	6.87
$T_{decrypt}$	1.19	1.42	0.16	5.80	0.52	0.43	1.18	2.27	6.62
$T_{database}$	7.05	2.98	1.02	24.20	5.87	5.65	6.42	2.43	9.52

**Table 3:** Execution times of the fog gateway.

Time Component	Execution Time (ms)
$T_{fog}$	10.19
$T_{total}$	10.35

**Table 4:** Comparison of simulation results.

Feature	Architecture A	Architecture B	Architecture C
Average execution time	209.96 ms	Similar to arch. A	19.44 ms
BCET	209.81 ms	Similar to arch. A	10.28 ms
WCET	217.78 ms	Similar to arch. A	210.41 ms

**Table 5:** Approximation of simulation results.

Feature	Architecture A	Architecture B	Architecture C
Average execution time	21·T	21·T + $T_{link}$	2·T
BCET	21·T	21·T + $T_{link}$	*T*
WCET	22·T	22·T + $T_{link}$	21·T

**Table 6:** The simulation results for different workloads.

Fog Gateway Utilization	Architecture A	Architecture B	Architecture C
33%	10.42 ms	214.16 ms	213.30 ms
66%	15.93 ms	219.62 ms	217.57 ms
99%	24.50 ms	252.48 ms	248.81 ms
